# Inhibition of β-catenin signaling attenuates arteriovenous fistula thickening in mice by suppressing myofibroblasts

**DOI:** 10.1186/s10020-022-00436-1

**Published:** 2022-01-21

**Authors:** Chung-Te Liu, Shih-Chang Hsu, Hui-Ling Hsieh, Cheng-Hsien Chen, Chun-You Chen, Yuh-Mou Sue, Tso-Hsiao Chen, Yung-Ho Hsu, Feng-Yen Lin, Chun-Ming Shih, Yan-Ting Shiu, Po-Hsun Huang

**Affiliations:** 1 Graduate Institute of Clinical Medicine, College of Medicine, Taipei Medical University, Taipei, Taiwan; 2 Division of Nephrology, Department of Internal Medicine, Wan-Fang Hospital, Taipei Medical University, Taipei, Taiwan; 3 Department of Internal Medicine, School of Medicine, College of Medicine, Taipei Medical University, Taipei, Taiwan; 4 TMU Research Center of Urology and Kidney, Taipei Medical University, Taipei, Taiwan; 5 Emergency Department, Department of Emergency and Critical Medicine, Wan-Fang Hospital, Taipei Medical University, Taipei, Taiwan; 6 Department of Emergency Medicine, School of Medicine, College of Medicine, Taipei Medical University, Taipei, Taiwan; 7 Graduate Institute of Medical Science, National Defense Medical Center, Taipei, Taiwan; 8 Division of Nephrology, Department of Internal Medicine, Shuang-Ho Hospital, Taipei Medical University, New Taipei City, Taiwan; 9 Department of Radiation Oncology, Wan-Fang Hospital, Taipei Medical University, Taipei, Taiwan; 10 Division of Nephrology, Department of Internal Medicine, Shuang Ho Hospital, Taipei Medical University, New Taipei City, Taiwan; 11 Division of Cardiology and Cardiovascular Research Center, Department of Internal Medicine, Taipei Medical University Hospital, Taipei, Taiwan; 12 Division of Nephrology and Hypertension, University of Utah, 295 Chipeta Way, Suite 4000, Salt Lake City, UT 84109 USA; 13 Veterans Affairs Medical Center, Salt Lake City, UT USA; 14 Division of Cardiology, Department of Medicine, Taipei Veterans General Hospital, 112, No. 201, Sec. 2, Shih-Pai Road, Taipei, Taiwan; 15 Cardiovascular Research Center, National Yang-Ming University, Taipei, Taiwan; 16 Institute of Clinical Medicine, National Yang-Ming University, Taipei, Taiwan

**Keywords:** Arteriovenous fistula (AVF), β-catenin, Hemodialysis, Myofibroblast

## Abstract

**Background:**

Arteriovenous fistula (AVF) is the most important vascular access for hemodialysis; however, preventive treatment to maintain the patency of AVFs has not been developed. In endothelium, β-catenin functions in both the intercellular adherens complex and signaling pathways that induce the transition of endothelial cells to myofibroblasts in response to mechanical stimuli. We hypothesize that mechanical disturbances in the AVF activate β-catenin signaling leading to the transition of endothelial cells to myofibroblasts, which cause AVF thickening. The present study aimed to test this hypothesis.

**Methods:**

Chronic kidney disease in mice was induced by a 0.2% adenine diet. AVFs were created by aortocaval puncture. Human umbilical vein endothelial cells (HUVECs) were used in the cell experiments. A pressure-culture system was used to simulate mechanical disturbances of the AVF.

**Results:**

Co-expression of CD31 and smooth muscle alpha-actin (αSMA), loss of cell–cell adhesions, and the expression of the myofibroblast marker, integrin subunit β6 (ITGB6), indicated transition to myofibroblasts in mouse AVF. Nuclear translocation of β-catenin, decreased axin2, and increased c-myc expression were also observed in the AVF, indicating activated β-catenin signaling. To confirm that β-catenin signaling contributes to AVF lesions, β-catenin signaling was inhibited with pyrvinium pamoate; β-catenin inhibition significantly attenuated AVF thickening and decreased myofibroblasts. In HUVECs, barometric pressure-induced nuclear localization of β-catenin and increased expression of the myofibroblast markers, αSMA and ITGB6. These changes were attenuated via pretreatment with β-catenin inhibition.

**Conclusions:**

The results of this study indicate that mechanical disturbance in AVF activates β-catenin signaling to induce the transition of endothelial cells to myofibroblasts. This signaling cascade can be targeted to maintain AVF patency.

**Supplementary Information:**

The online version contains supplementary material available at 10.1186/s10020-022-00436-1.

## Introduction

Arteriovenous fistula (AVF) is the recommended vascular access for patients on maintenance hemodialysis because of superior outcomes (Brown et al. 2017). Nevertheless, the suboptimal patency rate is a significant clinical issue. The 1 year primary patency rates for newly created AVFs are 50–70% (Al-Jaishi et al. 2014; Schinstock et al. 2011; Huijbregts et al. 2008; Field et al. 2008). In 2017, healthcare expenditures for maintaining AVF patency were higher than expenditures for any other procedures in patients with end-stage renal disease in Taiwan; the expenditures for maintaining AVFs were more than 1.2 billion U.S. dollars (Annual Report on Kidney Disease in Taiwan 2019). Despite the low AVF patency rates, current strategies for maintaining AVFs are limited to blood flow monitoring and early angioplasty (Lok et al. 2020). Preventive strategies for maintaining AVF patency are needed.

Cell co-expressing smooth muscle alpha-actin (αSMA) and vimentin, known as myofibroblast, is one of the main causes of AVF patency loss (Roy-Chaudhury et al. 2007). The formation of AVF thickening impairs outward remodeling and ultimately leads to stenosis and patency loss (Roy-Chaudhury et al. 2006). The mechanisms leading to AVF thickening are controversial. One proposed mechanism is the activation of Notch signaling or transforming growth factor-β, which leads to the transition and migration of myofibroblasts (Liang et al. 2015; Brahmbhatt et al. 2016). Among the various signaling pathways that cause the transition to myofibroblasts, β-catenin signaling has been extensively studied in embryology and oncology research. Despite the significance of myofibroblasts in AVF thickening, the role of β-catenin signaling in the pathogenesis of AVF thickening has rarely been mentioned (Gong et al. 2017).

In endothelial cells, β-catenin localizes to the cell membrane as a component of the intercellular adherens complex to help maintain the integrity of the endothelium (Guo et al. 2008). According to an in vitro study, when the intercellular junction is disturbed, β-catenin moves from the plasma membrane to the nucleus (Tharakan et al. 2012). In the aortas of ApoE − / − mice, increased shear stress activated β-catenin signaling in atherosclerotic lesions and upregulated the expression of the fibrosis marker, fibronectin (Gelfand et al. 2011). Additionally, β-catenin signaling activates endothelial-to-mesenchymal transition during heart cushion development, which involves the induction of αSMA and other mesenchymal markers in endothelial cells (Liebner et al. 2004). Mechanical disturbances and increased transition to myofibroblasts are also features of AVF thickening; thus, the activation of β-catenin signaling may play role in the formation of AVF thickening.

Based on the previous research, we hypothesized that mechanical disturbance of the AVF activates β-catenin signaling in the endothelium, leading to the transition of endothelial cells to myofibroblasts and the development of AVF thickening. In this study, a mouse AVF model and a pressurized cell model were used to test this hypothesis.

## Materials and methods

### Animal experiments

All animal experiments were approved by the Wan-Fang Hospital Animal Care and Use Committee (WAN-LAC-108-002). Male C57BL/6 mice aged 7 weeks (purchased from National Laboratory Animal Center, Taipei, Taiwan) were used for all animal experiments. After a week of adaptation, baseline plasma creatinine levels were measured to define baseline renal function. From 8 weeks old until the time of euthanasia, the mice were fed a 0.2% adenine diet (LabDiet, St. Louis, MO, USA) to induce chronic kidney disease (CKD) (Additional file 1: Fig. S1). AVF showed significantly wall thickening, where sham operated inferior vena cava (IVC) was used as the negative control. The presence of CKD significantly increased AVF thickening (Additional file 1: Fig. S2A–D, I) and the expression of αSMA (Additional file 1: Fig. S2E–H, J). Both sham operated mice and mice with AVF were fed the adenine diet. Plasma creatinine levels were measured after 8 weeks of adenine diet to confirm the renal impairment. AVF was induced at that time. Mice were sacrificed on weeks 2, 4, and 6 after AVF creation, depending on the experiment. The sham group was sacrificed 6 weeks after the sham surgery.

AVFs were created using aortocaval puncture. An AVF forms between the abdominal aorta and IVC in this model, which recapitulates hemodialysis AVF in humans (Yamamoto et al. 2013). Before surgery, mice received appropriate anesthesia and the abdominal skin was shaved and sterilized. The abdominal wall was opened from the lower end of the sternum to the upper end of pubic symphysis along the linea alba. The intestine was exteriorized to expose the retroperitoneal anatomy. The intestine was covered with wet gauze during the surgery. The abdominal aorta was identified and clamped inferior to the bifurcation of the left renal artery. The puncture site was premarked with a 5–0 nylon suture in the adjacent soft tissue. The aortocaval puncture was performed with a 25G needle. Bleeding was controlled by covering the puncture site with intestine or peritoneal fat tissue after removing the needle. If the AVF creation was successful, the dark-colored IVC changed to a light red color similar to the abdominal aorta, and pulsation of the IVC could be visualized. Doppler ultrasound was used to confirm the successful AVF creation. Finally, the intestine was returned to the abdomen, and the abdominal wall was closed.

Pyrvinium pamoate, which was used to inhibit β-catenin signaling, was dissolved in 4% dimethyl sulfoxide (DMSO) in saline and administered at 200 μg/kg/day by oral gavage. Oral gavage of 4% DMSO was used as a negative control for pyrvinium pamoate. Mice were treated with pyrvinium pamoate or DMSO from 1 week after operation to euthanasia.

### Specimen preparation, tissue sectioning, and histological methods

After euthanasia of mice, en bloc resection from the bifurcation of the common iliac veins to the lower end of the hepatic segment of the IVC was performed to include the IVC, abdominal aorta, underlying psoas muscles, and overlying peritoneum. The specimens were immediately fixed in paraformaldehyde and embedded in paraffin for tissue sectioning. Tissue was transversely sectioned at 3 μm at the puncture site, which had been premarked as stated previously. Additionally, the kidneys were resected for histological analyses to confirm the successful induction of CKD (Additional file 1: Fig. S3A–B). To identify AVF thickening, Masson trichrome staining was performed (Modified Masson’s Trichrome Stain Kit, ScyTek Laboratories). Periodic Acid-Schiff staining (PAS staining kit 101646, Merck Millipore) was used to evaluate renal histology. Immunohistochemistry (IHC) was performed using an Ultravision Quanto Detection System (Thermo Scientific). For IHC and tissue immunofluorescence (IF), the following primary antibodies were used: αSMA (Abcam, ab7817), calponin (Cell Signaling, #17819), CD31 (Abcam, ab28364), vimentin (Cell Signaling, 5741), integrin subunit β6 (ITGB6, Bioss, bs-5791R), β-catenin (Cell Signaling, 8480), axin2 (Abcam, ab32197), c-myc (Abcam, ab32072), Tie2 (Bioss, bs-1300R). The secondary antibodies were antirabbit IgG (Sigma-Aldrich, SI-SAB4600045) and antimouse IgG (Sigma-Aldrich, SI-SAB4600161). The isotype control IgG-stained cells and sections were shown in Additional file 1: Fig. S5, which yielded no signals when examined under conditions identical to those used for the protein-specific primary IgG.

Histologic images were obtained using a TissueFAXS system (TissueGnostics, Vienna, Austria). IF images were obtained and merged using a TissueFAXS system (TissueGnostics, Vienna, Austria) or Stellaris 8 confocal microscope (Leica Microsystems, Wetzlar, Germany). For confocal microscopic imaging, the optical slice thickness was 0.5 μm. DAB expression in IHC images was automatically analyzed using HistoQuest software. The expression of fluorescence in IF images was automatically analyzed using TissueQuest software. AVF thickening was quantitated by the maximal thickness of Masson Trichrome staining. Nuclear localization was defined on serial confocal microscope images, in which the target object surrounded by the signal of dapi suggests its nuclear localization. Analyses of the images were performed by a specialized technician in blinded manner.

### Cell culture and pressure–culture system

Human umbilical vein endothelial cells (HUVECs) (Bioresource Collection and Research Center, Hsinchu, Taiwan) from passages 2 to 5 were used. The cell experiments were conducted in an incubator with supplementary carbon dioxide (5% balanced by air). A pressure–culture system was used to simulate the effects of mechanical disturbance in AVF. In this system, HUVECs cultivated in tissue culture flasks were continuously subjected to barometric pressures of 50–70 mmHg for 4 h during the experiment, whereas the nonpressurized groups were cultured in flasks under normal pressures. For the inhibition of β-catenin signaling, HUVECs were pretreated with pyrvinium pamoate for 30 min before pressurization. Cells were treated with 100 nM final concentration of pyrvinium pamoate dissolved in 4% DMSO. Controls were treated with 4% DMSO only.

### Cell sample processing and western blotting

Cells were collected immediately after pressurization and stored at − 80 °C. Cells were lysed in RIPA Lysis Buffer and fractionated with a Membrane, Nuclear & Cytoplasmic Protein Extraction Kit (Bio Basic #BSP002). For western blotting, 20 μg of protein was loaded per well. Chemiluminescence (Bio-Rad, Clarity Western ECL, Substrate, catalog number: 1705061) was used to detect the signals. The following primary antibodies were used: αSMA (Abcam, ab7817), ITGB6 (Bioss, bs-5791R), glyceraldehyde 3-phosphate dehydrogenase (GAPDH, Cell Signaling, 2118), β-catenin (Cell Signaling, 8480), and lamin B1 (Abcam, ab16048). The following secondary antibodies were used: antirabbit IgG (Proteintech, SA 00001-2) and antimouse IgG (Proteintech, SA 00001-1).

### Statistics

Continuous variables were expressed as medians, first and third quartiles, and minimum and maximum values as shown in boxplots. Statistical comparisons were made using the Wilcoxon signed-rank test. Bonferroni correction applied in cases of multiple comparisons, such that statistical significance was defined as a P value < 0.05/number of hypotheses tested. All data analyses were conducted using SAS 9.4 software (SAS Institute, Cary, NC, USA). All data used in this study are available from the corresponding author for research purposes.

## Results

### Transition to myofibroblasts in mouse AVF

**Fig. 1:**
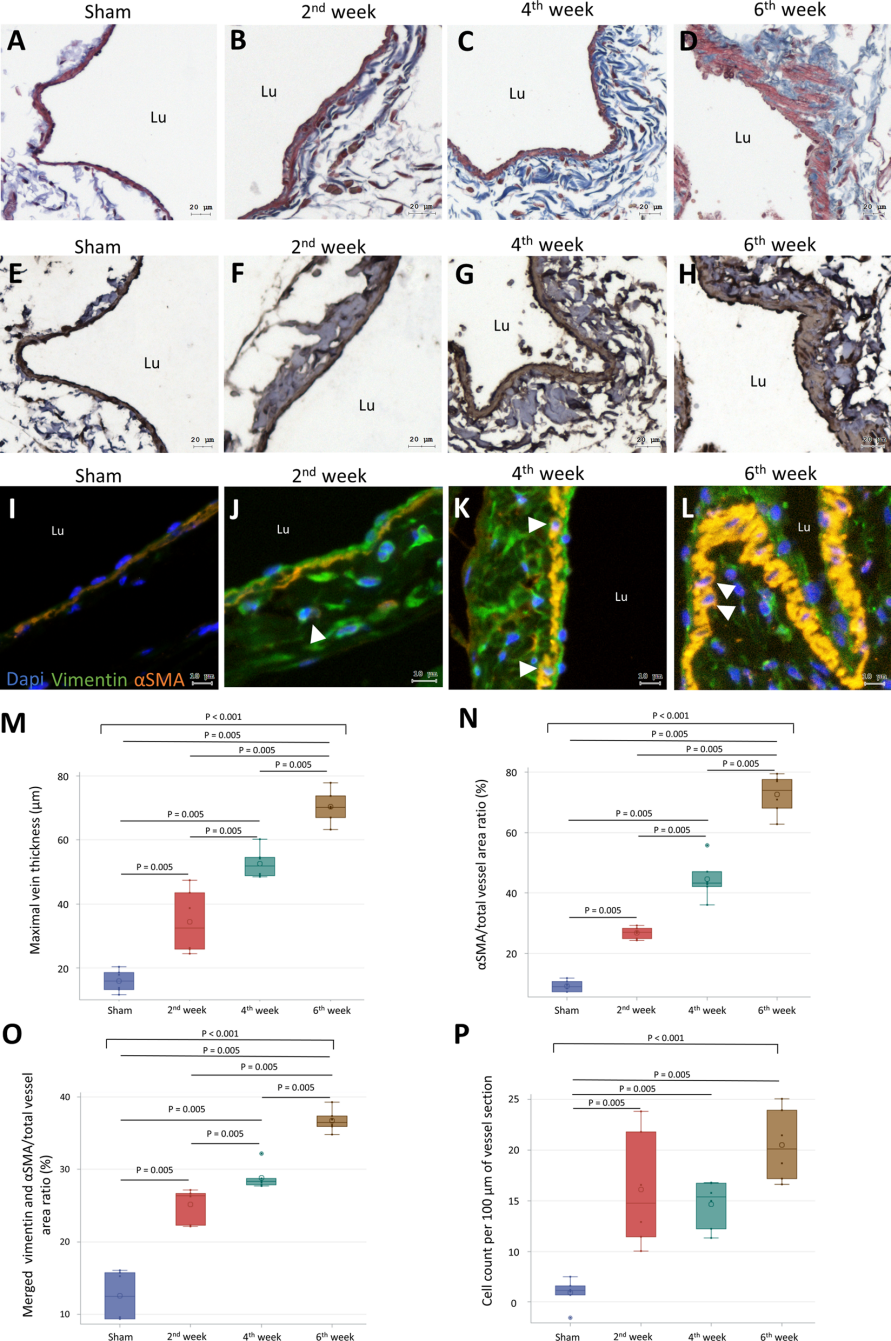
Increased myofibroblast in mouse AVF thickening. Sham operated IVC specimens were obtained 6 weeks post-operation. AVF specimens were obtained at 2, 4, and 6 weeks post-operation. **A–D** Masson trichrome stained sections. **E–H** Expression of αSMA shown by IHC. **I–L** IF images double stained for vimentin and αSMA (arrowhead, double-positive cells). **M** Quantification of vein thickness measured in Masson trichrome stained sections. **N** Quantification of αSMA expression measured in IHC sections. **O** Quantification of the αSMA/vimentin merged area measured in IF images. **P** Quantification of αSMA/vimentin double-positive cells in tunica media. Data are presented as median, 1st and 3rd quartiles, maximal and minimal values in the box plots (n = 6 per group). P values in **M–P** were determined by the Wilcoxon signed-rank test with Bonferroni correction. Insignificant P values are not shown. Images were obtained using the TissueFAXS system. *CKD* chronic kidney disease, *AVF* arteriovenous fistula, *IVC* inferior vena cava, *IHC* immunohistochemistry, *αSMA* smooth muscle alpha-actin, *IF* immunofluorescence, *Lu* lumen

To examine the process of AVF thickening, AVF specimens were obtained at 2, 4, and 6 weeks post-operation. Sham operated IVC was used as the negative control (n = 6 per group). The tunica media of sham operated IVC contained a single layer of smooth muscle cells (Fig. 1A). In contrast, AVF specimens showed thickened tunica media composed of multiple layers of cells and increased collagen (Fig. 1B–D). Compared with sham operated IVC, AVF thickness increased significantly from week 2 to week 6 post-operation (Fig. 1M). Within the thickened vessel walls of AVF, the expression of αSMA significantly increased from week 2 to week 6 post-operation, suggesting altered vascular remodeling in AVF (Fig. 1E–H, N). Nevertheless, the thickened AVF wall rarely expressed calponin, suggesting that the proliferation of vascular smooth muscle cells was not the main cause of AVF thickening (Additional file 1: Fig. S4). The specimens were double stained for αSMA and vimentin to confirm the origin of increased αSMA in AVF. In sham operated IVC, vimentin expression and a double-positive stained area were rarely observed (Fig. 1I). In the tunica media of AVF, the double-positive stained area for αSMA and vimentin increased significantly from week 2 to week 6 post-operation. Cells double-positive for αSMA and vimentin were observed in the same area, suggesting myofibroblasts (Fig. 1J–L). Compared with sham operated IVC, AVF showed significantly increased αSMA/vimentin double-positive area and cells (Fig. 1O–P). Taken together, these findings suggest that increased myofibroblasts contribute to AVF thickening.

### Mesenchymal transition of endothelial cells in mouse AVF

**Fig. 2:**
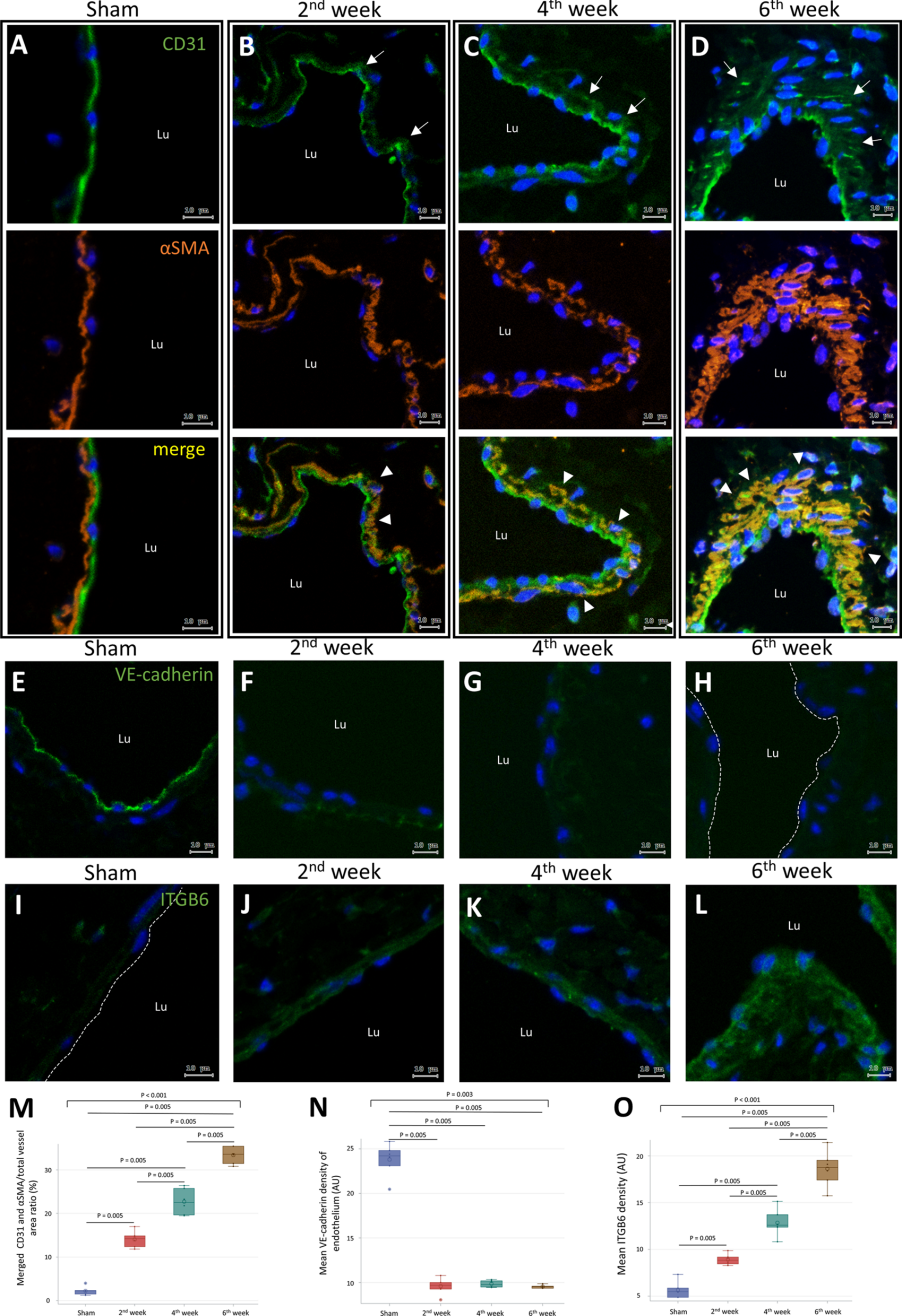
The mesenchymal transition of endothelial cells in mouse AVF thickening. Sham operated IVC specimens were obtained 6 weeks post-operation. AVF specimens were obtained at 2, 4, and 6 weeks post-operation. **A–D** IF images double stained for CD31 and αSMA (arrows, expression of CD31 in tunica media; arrowhead, double-positive cells). **E–H** IF images stained for VE-cadherin (dotted line, luminal surface of endothelium). **I–L** IF images stained for ITGB6 (dotted line, luminal surface of endothelium) **M** Quantification of the CD31/αSMA double-positive area. **N** Quantification of VE-cadherin expression. **O** Quantification of ITGB6 expression. Data are presented as median, 1st and 3rd quartiles, maximal and minimal values in the box plots (n = 6 per group). P values in **M–O** were determined by the Wilcoxon signed-rank test with Bonferroni correction. Insignificant P values are not shown. Images were obtained using the TissueFAXS system. *AVF* arteriovenous fistula, *IVC* inferior vena cava, *IF* immunofluorescence, *αSMA* smooth muscle alpha-actin, *VE-cadherin* vascular endothelial-cadherin, *ITGB6* integrin subunit β6, Arbitrary unit (AU) = integrated density/area

To investigate the origin of myofibroblasts in AVF thickening, sham operated IVC and AVF were double stained for CD31 and αSMA to determine if the myofibroblasts carried the endothelial marker CD31. In sham operated IVCs, both CD31 and αSMA distributed linearly with rare overlap, demonstrating the discriminating ability of these two markers for endothelial and mesenchymal cells (Fig. 2A). In AVF sections, co-expressed αSMA and CD31 in the tunica media could be identified since the 2nd week after AVF creation (Fig. 2B–D). As tunica media of the AVF thickened over time, the CD31/αSMA co-expression area also increased significantly (Fig. 2M).

VE-cadherin was evaluated as a marker of endothelial cell–cell adhesion. The endothelium of sham operated IVCs expressed VE-cadherin linearly and strongly (Fig. 2E). The expression of VE-cadherin decreased significantly in the AVF endothelium, suggesting the loss of cell–cell adhesions (Fig. 2F–H, N). To confirm the presence of myofibroblasts in AVFs, sham operated IVCs and AVFs were stained for ITGB6, which is a marker of fibrosis and the transition to myofibroblasts (Liu et al. 2021; Tatler et al. 2016; Khan and Marshall 2016). In sham operated IVCs, ITGB6 was rarely expressed in the intima and media (Fig. 2I). In the vascular wall of AVFs, the expression of ITGB6 increased significantly in both the intima and media (Fig. 2J–L, O).

**Fig. 3:**
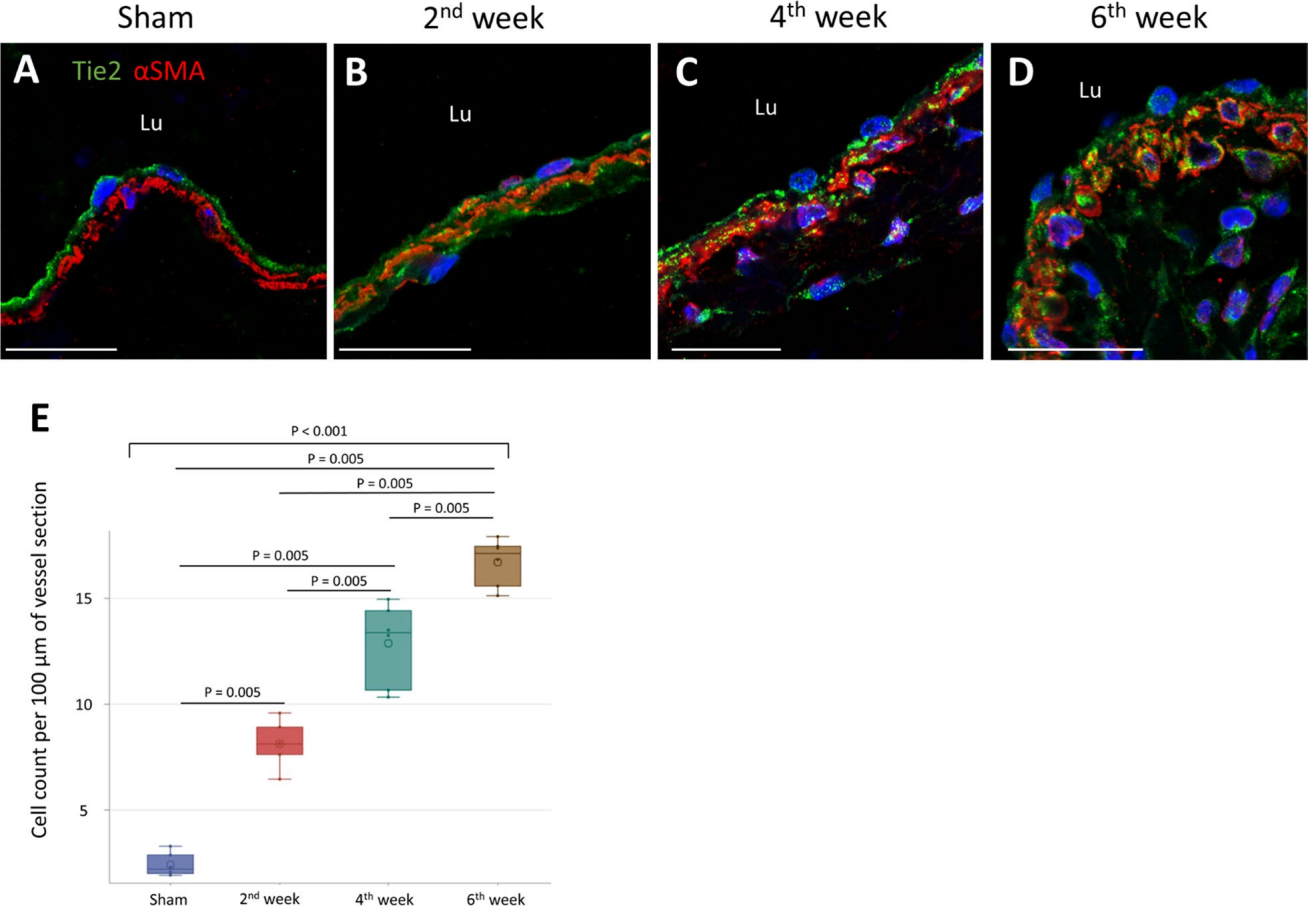
Expression of the endothelial marker Tie2 in myofibroblasts of AVF. Sham operated IVC specimens were obtained at 6 weeks post-operation. AVF specimens were obtained at 2, 4, and 6 weeks post-operation. **A–D** IF images double stained for Tie2 and αSMA (arrowhead, double-positive cells in tunica media; scale bar = 10 μm). **E** Quantification of Tie2/αSMA double-positive cells in tunica media. Data are presented as median, 1st and 3rd quartiles, maximal and minimal values in the box plots (n = 6 per group). P values were determined by the Wilcoxon signed-rank test with Bonferroni correction. Insignificant P values are not shown. Images were obtained by confocal microscopy. *AVF* arteriovenous fistula, *IVC* inferior vena cava, *IF* immunofluorescence, *αSMA* smooth muscle alpha-actin, *Lu* lumen

To confirm the origin of myofibroblasts, double staining for Tie2, an endothelial marker, and αSMA was performed. IF images were then obtained using a confocal microscope to confirm their co-localization. In the sham operated mice, cells double-positive for Tie2 and αSMA were rarely observed (Fig. 3A). In contrast, in the AVF sections, double-positive cells were readily identified in the tunica media (Fig. 3B–D). The double-positive stained cells in the AVF tunica media increased continuously from week 2 to week 6 post-operation (Fig. 3E). Overall, these results suggest that the transition to myofibroblasts increases in the AVF vascular wall and that the myofibroblasts are likely transitioning from endothelial cells.

### Activated β-catenin signaling in mouse AVF

**Fig. 4:**
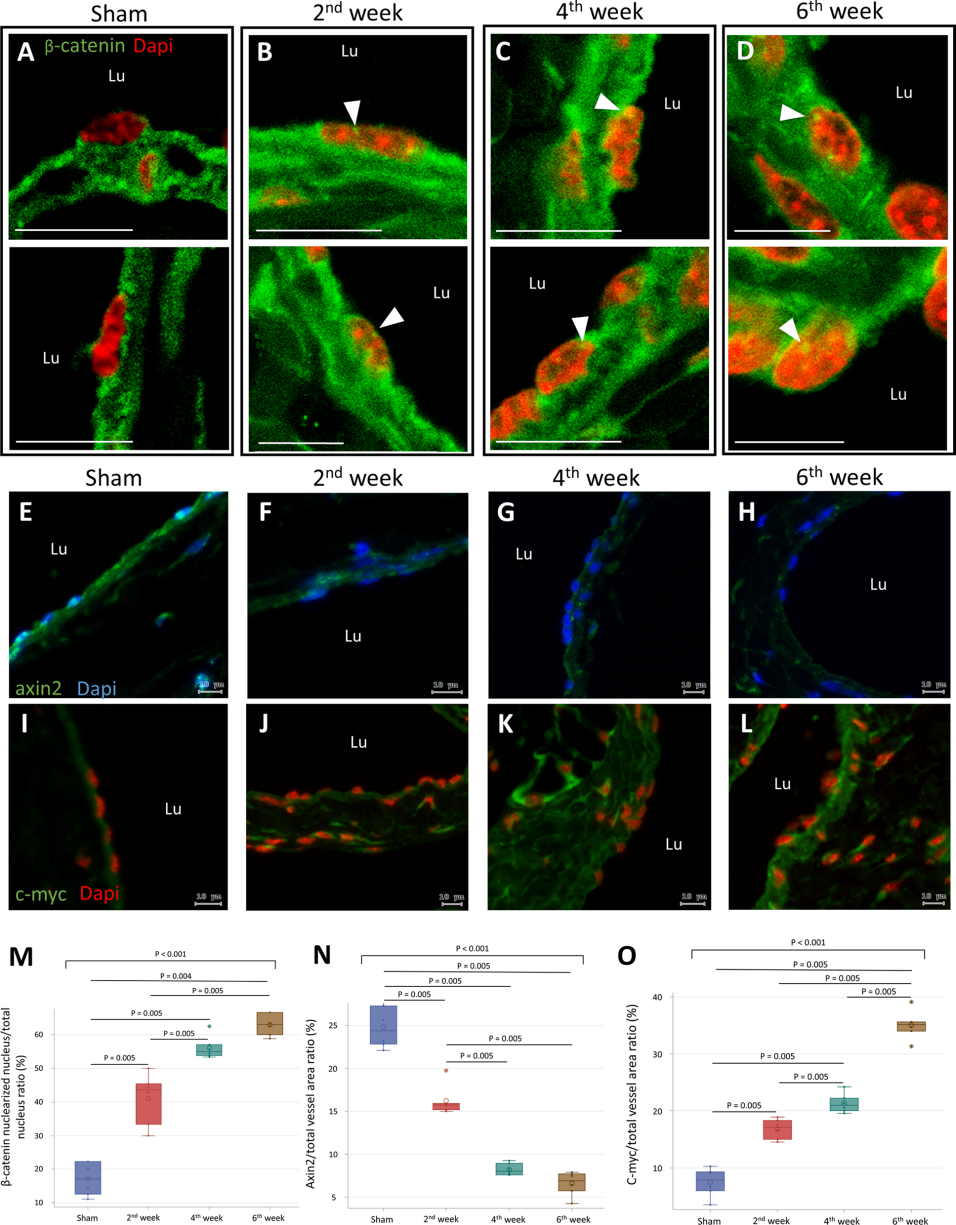
β-catenin signaling in mouse AVF thickening. Sham operated IVC specimens were obtained at 6 weeks post-operation. AVF specimens were obtained at 2, 4, and 6 weeks post-operation. **A–D** Nuclearization of β-catenin in endothelial cells is shown by merged green/red IF in the nuclei. The images are representative of n = 6 experiments (arrowhead, nucleus with β-catenin localization; scale bar = 10 μm). **E–H** IF images stained for axin2. **I–L** IF images stained for c-myc. **M** Quantification of β-catenin nuclearization. **N** Quantification of axin2 expression. **O** Quantification of c-myc expression. Data are presented as median, 1st and 3rd quartiles, maximal and minimal values in the box plots (n = 6 per group). P values in **M–O** were determined by the Wilcoxon signed-rank test with Bonferroni correction. Insignificant P values are not shown. Images in **A–D** were obtained by confocal microscopy. Images in **E–L** were obtained using the TissueFAXS system. *AVF* arteriovenous fistula, *IVC* inferior vena cava, *Lu* lumen

When β-catenin signaling is activated, the β-catenin destruction complex decomposes and β-catenin localizes to the nucleus to initiate cell transition. Therefore, we assessed the nuclearization of β-catenin, expression of axin2 (a component of the β-catenin destruction complex), and c-myc (a protein positively regulated by β-catenin signaling) in mouse AVF. In sham operated IVC, β-catenin was expressed in the endothelium, although nuclear localization was rarely observed (Fig. 4A). In AVF endothelium, nuclear localization of β-catenin was readily identified (Fig. 4B–D). Additionally, the frequency of β-catenin nuclearization significantly increased from week 2 to week 6 post-operation (Fig. 4M). In sham operated IVC, the expression of axin2 was readily observed in both the endothelium and tunica media (Fig. 4E). In the AVFs, the expression of axin2 decreased significantly from week 2 to week 6 post-operation (Fig. 4F–H, N). In contrast to axin2, the expression of c-myc increased significantly from week 2 to week 6 post-operation (Fig. 4I–L, O). These results suggest that β-catenin signaling was activated in mouse AVF.

### AVF myofibroblasts were attenuated by inhibition of β-catenin signaling

**Fig. 5:**
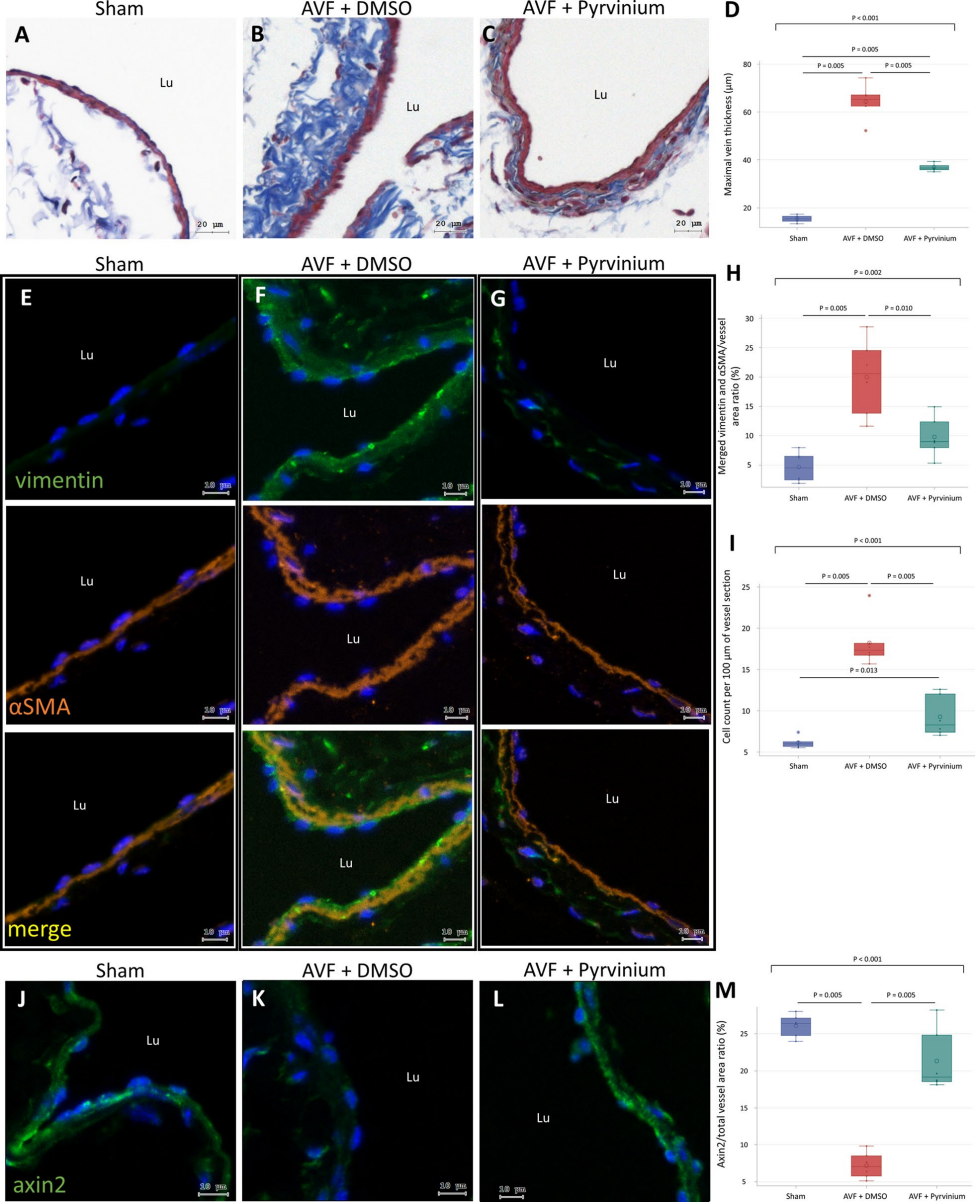
The effect of β-catenin inhibition on mouse AVF thickening. Treatment of pyrvinium pamoate or control solvent DMSO was started 1 week post-operation. Sham operated IVC and AVF specimens were obtained at 6 weeks post-operation. **A–C** Masson trichrome stained sections **D** Quantification of vein thickness measured in Masson trichrome stained sections. **E–G** IF images double-stained for αSMA and vimentin. **H** Quantification of αSMA/vimentin merged area **I** Quantification of αSMA/vimentin double-positive cells in tunica media. **J–L** IF images stained for axin2. **M** Quantification of axin2 expression. Data are presented as median, 1st and 3rd quartiles, maximal and minimal values in the box plots (n = 6 per group). P values in **D, H, I**, and** M** were determined by the Wilcoxon signed-rank test with Bonferroni correction. Insignificant P values are not shown. Images were obtained using the TissueFAXS system. *AVF* arteriovenous fistula, *IVC* inferior vena cava, *DMSO* dimethyl sulfoxide, *IF* immunofluorescence, *αSMA* smooth muscle alpha-actin, *Lu* lumen

To confirm the contributing role of β-catenin signaling in AVF thickening, mice receiving AVF creation were treated with pyrvinium pamoate, an anthelmintic drug with an inhibitory effect on β-catenin signaling (Li et al. 2021; Polosukhina et al. 2017). Mice were divided into the following three groups: a sham operated group; a group with AVF treated with the control solvent, DMSO; and a group with AVF treated with pyrvinium pamoate (n = 6 per group). As observed in the previous experiment, sham operated IVC showed thin tunica media composed of a single layer of smooth muscle cells, whereas AVF treated with DMSO showed significantly thickened tunica media. In AVF treated with pyrvinium pamoate, tunica media thickening was attenuated compared to AVF treated with DMSO (Fig. 5A–D). Vimentin and αSMA double staining indicated weak expression of vimentin in sham operated IVCs (Fig. 5E). In AVF treated with DMSO, the expression of vimentin increased and double-positive myofibroblasts were readily observed (Fig. 5F). In AVF from mice treated with pyrvinium pamoate, the expression of vimentin was attenuated and myofibroblasts decreased significantly compared with AVF treated with DMSO (Fig. 5G–I).

The expression of axin2 was used to confirm the inhibition of β-catenin signaling by pyrvinium pamoate. As observed in the previous experiment, sham operated IVCs strongly expressed axin2. In AVF treated with DMSO, the expression of axin2 decreased significantly. In AVF treated with pyrvinium pamoate, the expression of axin2 increased significantly compared with AVF treated with DMSO (Fig. 5J–M). These results suggest that activated β-catenin signaling contributes to increased myofibroblasts in AVF.

### Barometric pressure-activated β-catenin signaling induces endothelial cell transition to myofibroblasts

**Fig. 6:**
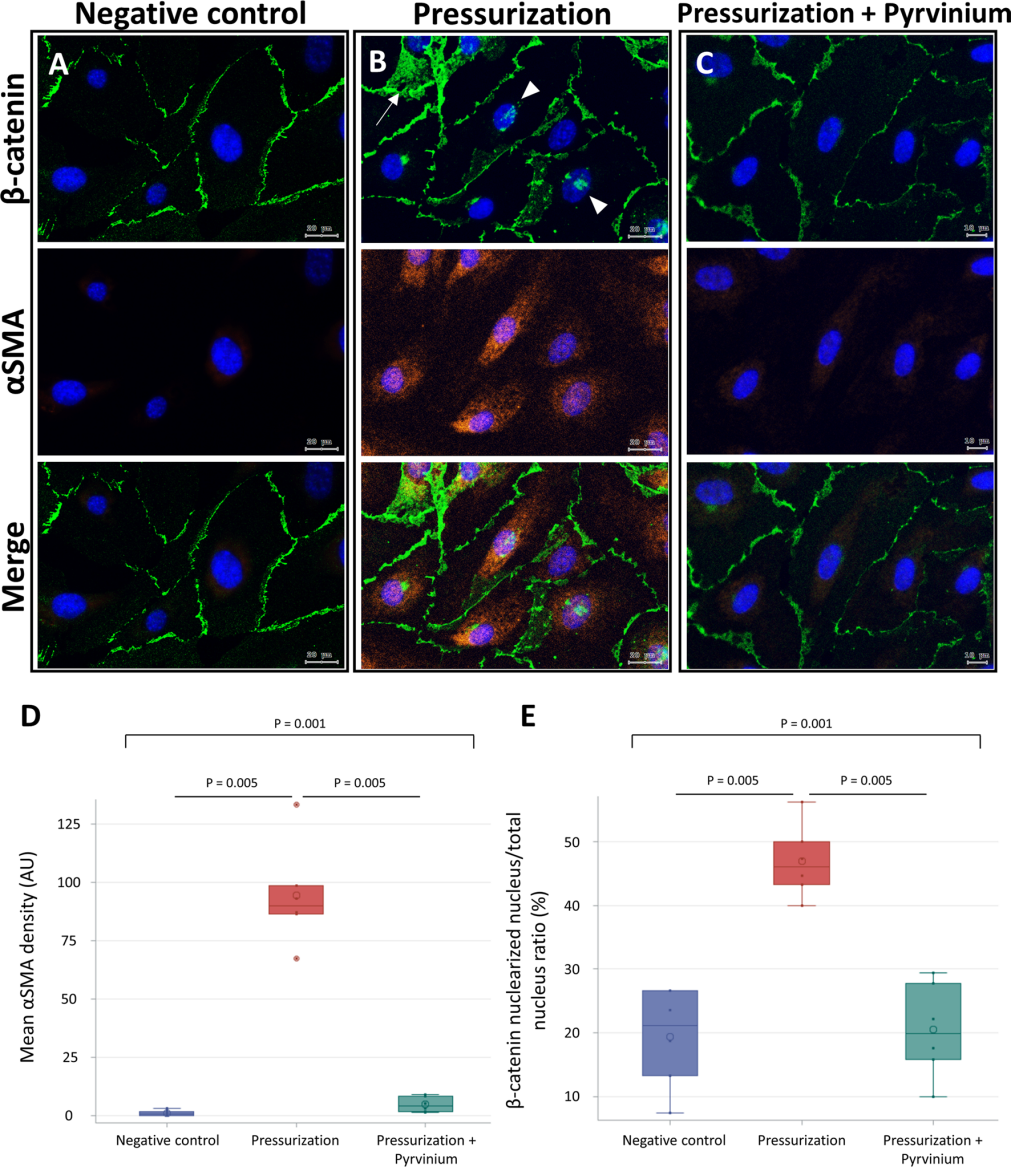
β-catenin disturbance and mesenchymal transition of HUVEC with increased barometric pressure. HUVECs exposed to increased barometric pressure were treated with control solvent DMSO or pyrvinium pamoate. The negative control group received DMSO treatment only. **A–C** IF images double stained for β-catenin and αSMA. **D** Quantification of αSMA expression. **E** Quantification of β-catenin nuclearization. Data are presented as median, 1st and 3rd quartiles, maximal and minimal values in the box plots (n = 5 per group). P values in **D, E** were determined by the Wilcoxon signed-rank test with Bonferroni correction. Insignificant P values are not shown. Images were obtained using the TissueFAXS system. *HUVEC* human umbilical endothelial venous cell, *DMSO* dimethyl sulfoxide, *αSMA* smooth muscle alpha-actin, Arbitrary unit (AU) = integrated density/area

To investigate the effect of mechanical disturbance on cell transition, HUVECs were cultivated in a standard incubator or pressure-culture system and then separated into three experimental groups as follows: a negative control group with no barometric pressurization, a group treated with barometric pressure, and a group pretreated with pyrvinium pamoate followed by barometric pressure. In the negative control group, β-catenin was distributed linearly along the cell membrane. Cytoplasmic distribution and nuclear localization of β-catenin were absent. No αSMA expression was observed in the negative control group (Fig. 6A). In pressurized cells, membranous β-catenin was distributed in the cytoplasm and nucleus. The expression of αSMA was induced by increased barometric pressure, and the αSMA exhibited a spindle-shaped pattern similar to mesenchymal cells (Fig. 6B). In pressurized HUVECs pretreated with pyrvinium pamoate, both β-catenin nuclear localization and the expression of αSMA were significantly attenuated (Fig. 6C–E).

**Fig. 7:**
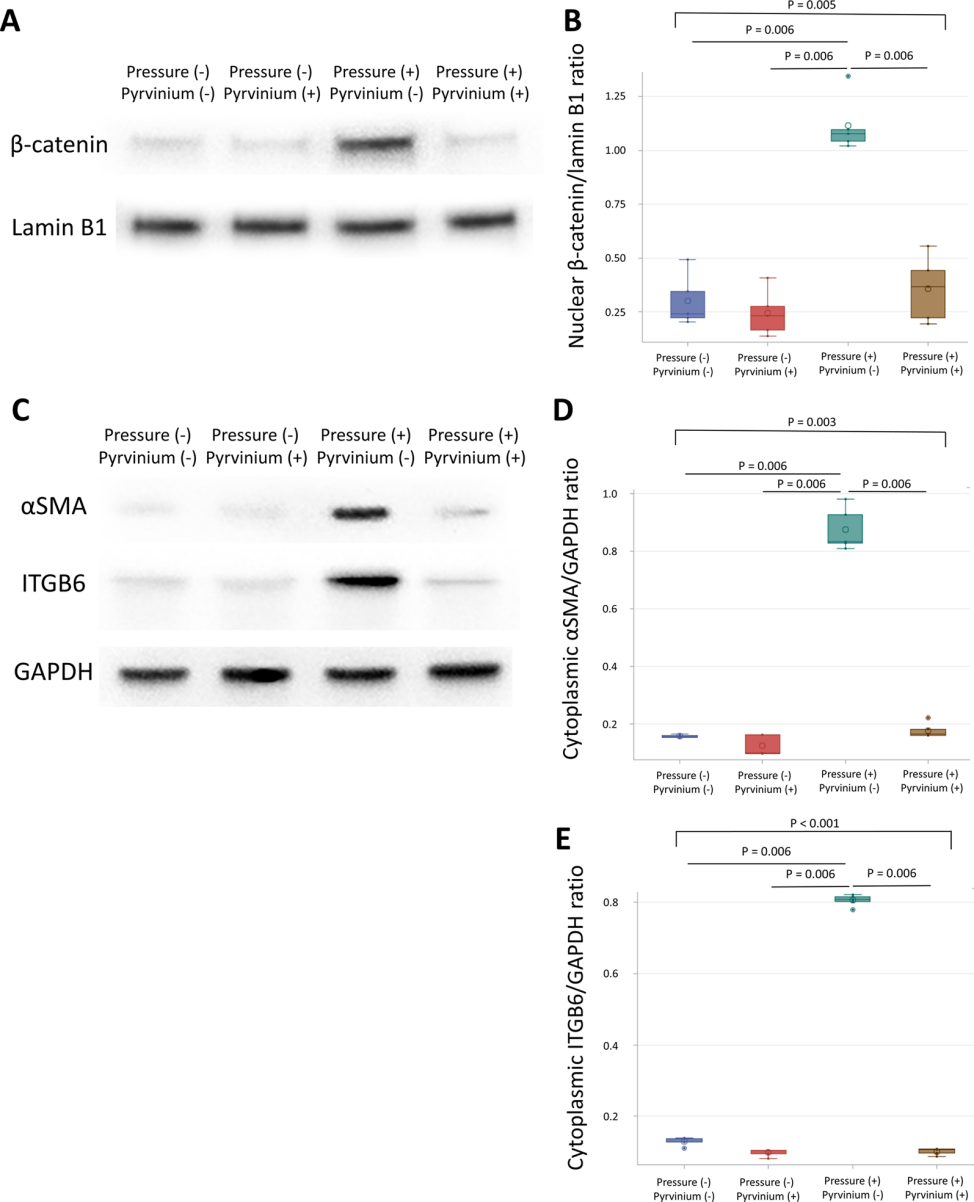
Western blot of nuclear β-catenin and cytoplasmic myofibroblast markers of HUVEC with increased barometric pressure. Pressurized or nonpressurized HUVECs were treated with control solvent DMSO or pyrvinium pamoate to create the four experimental groups. **A** Western blots of nuclear protein extracts for β-catenin. Lamin B1 served as the loading control. **B** Quantification of nuclear β-catenin. **C** Western blots of cytoplasmic protein extracts for αSMA and ITGB6. GAPDH served as the loading control. **D** Quantification of cytoplasmic αSMA. **E** Quantification of cytoplasmic ITGB6. Data are presented as median, 1st and 3rd quartiles, maximal and minimal values in the box plots (n = 5 per group). P values in **B**, **D**, and** E** were determined by the Wilcoxon signed-rank test with Bonferroni correction. Insignificant P values are not shown. *HUVEC* human umbilical endothelial venous cell, *DMSO* dimethyl sulfoxide, *αSMA* smooth muscle alpha-actin, *ITGB6* integrin subunit β6, *GAPDH* glyceraldehyde-3-phosphate dehydrogenase

Western blotting was used to confirm the effects of barometric pressure on β-catenin signaling and the transition to myofibroblasts. HUVECs were divided into four treatment groups as follows: nonpressurized, no pyrvinium treatment; nonpressurized, pyrvinium-treated; pressurized, no pyrvinium treatment; and pressurized, pyrvinium-treated. The pressurization protocol and pyrvinium treatment were the same as in the previous experiment. Western blotting showed that barometric pressure significantly increased nuclear β-catenin protein expression, and treatment with pyrvinium pamoate attenuated β-catenin nuclearization (Fig. 7A, B). Additionally, barometric pressure significantly increased cytoplasmic αSMA and ITGB6, which are myofibroblast markers, in HUVECs. The expression of these myofibroblast markers were significantly attenuated by pyrvinium pamoate (Fig. 7C–E). These results demonstrate that barometric pressure activated β-catenin signaling to induce the transition of HUVECs to myofibroblasts (the uncut Western blots are shown in Additional file 1: Fig. S5).

## Discussion

This study showed that activated β-catenin signaling in mouse AVF may contribute to the transition of endothelial cells to myofibroblasts, leading to the formation of AVF thickening. In mouse AVF, inhibition of β-catenin signaling significantly attenuated the transition to myofibroblasts and the thickening of AVF. Our cell experiments showed that barometric pressure activates β-catenin signaling and, consequently, induces the transition of HUVECs to myofibroblasts. Taken together, these results imply that mechanical disturbances activate β-catenin signaling to induce the transition of endothelial cells to myofibroblasts, which subsequently cause AVF thickening. Hence, β-catenin signaling is a potential target for the prevention of AVF thickening.

Increased αSMA-expressing mesenchymal cells have been a universal finding in different types of vascular remodeling. Although the source of these mesenchymal cells remains controversial, a body of evidence supports the endothelial cell as an origin of pathogenic mesenchymal cells in the process of vascular remodeling (Arciniegas et al. 2007). In a study by Cooley et al., they showed that the endothelial-to-mesenchymal transition contributes to neointimal formation in mouse vein grafts using lineage tracing transgenic mice (Cooley et al. 2014). In an in vitro study, human dermal microvascular endothelial cells transitioned to the mesenchymal phenotype in response to interleukin-1 stimulation (Romero et al. 1997). In an earlier study, the presence of undifferentiated endothelial cells in coronary allografts implicated the role of endothelial-to-mesenchymal transition in transplant atherosclerosis (Beranek and Cavarocchi 1990). Additionally, transition to mesenchymal cells occurs in hypoxia-induced pulmonary vascular remodeling (Zhu et al. 2006). In line with these previous studies, the present study also showed that endothelial cells may be an origin of myofibroblasts in pathogenic thickening of AVF.

Mechanical disturbances, including tractional or tensile forces, regulate cell adhesion, migration, and differentiation (Huang and Ingber 2000; Ingber 1993). Shear stress increases endothelial cell migration by modulating traction forces (Tzima 2006). Despite these previous studies, the mechanism by which mechanical forces regulate endothelial-to-mesenchymal transition is unclear (Arciniegas et al. 2007). β-catenin functions as both part of the intercellular adherens complex (Guo et al. 2008) and the cell signaling pathway that induces phenotypic transition (Gong et al. 2017); thus, β-catenin may play a central role in the crosstalk between mechanical disturbances and the transition to mesenchymal cells. In a study on epithelial-to-mesenchymal transition, transforming growth factor-β1 induced the loss of β-catenin association with E-cadherin and α-catenin to interrupt the integrity of cell-to-cell adhesions. This resulted in a morphologic change in epithelial cells, loss of cell–cell contacts, and cell migration. Mechanical disturbance may have a similar role in endothelial cells through β-catenin signaling (Tian and Phillips 2002). In the present study, we found clues to the endothelial transition to myofibroblasts in mouse AVF thickening. We found co-expression of endothelial and mesenchymal markers (CD31 and αSMA), decreased cell–cell adhesion (VE-cadherin), and increased myofibroblast markers, ITGB6. Furthermore, activated β-catenin signaling in AVF lesions was demonstrated by nuclear localization of β-catenin, decreased expression of axin2, and increased expression of c-myc. The cell experiments showed that barometric pressure induced nuclear localization of β-catenin and expression of αSMA and ITGB6 in HUVECs. In line with previous studies, our results suggest that mechanical signals in AVF activate β-catenin signaling to induce the transition to myofibroblasts and, subsequently, the development of AVF thickening.

Epithelial-to-mesenchymal transition is essentially reversible (Arciniegas et al. 2007). Bone morphogenic protein-7 can reverse epithelial-to-mesenchymal transition by upregulating the expression of cadherin (Okada and Kalluri 2005a, b). Recombinant human bone morphogenic protein-7 can also induce remission of fibrosis in kidneys with severe fibrosis (Zeisberg et al. 2003). Nevertheless, the reversibility of endothelial transition to myofibroblasts in AVF has not been investigated. In the present study, we demonstrated that pyrvinium pamoate attenuated mouse AVF thickening when administered early after AVF creation. However, pyrvinium pamoate should be administered at a later stage of AVF development to determine if pyrvinium pamoate reverses AVF thickening that are already formed.

Aiming for the same goal as the present study, Zhao et al. used a vascular smooth muscle cell lineage tracing reporter mouse to investigate the origins of AVF lesions (Zhao et al. 2017). They found severely damaged endothelium and that thickened medial layer of the AVF was mainly composed of differentiated vascular smooth muscle cells. Liang et al. investigated the origin of cells forming AVF thickening using transgenic mice whose neural crest-derived carotid arterial smooth muscle cells were labeled with green fluorescence. Their results showed that approximately 50% of αSMA-expressing cells were of arterial origin and that Notch activation was required for the migration of arterial smooth muscle cells into the lesion (Liang et al. 2015). Although these studies suggest that vascular smooth muscle is an important origin of AVF thickening, their findings also implied the complexity of lesion components and the possibility of multiple origins. The results of the present study do not imply that the origin of AVF myofibroblasts is exclusively from endothelial cells; rather, the results suggest that endothelial cells are one of the contributing sources and that activated β-catenin signaling is one of the pathogenic mechanisms.

Barometric pressure was used to simulate the mechanical signals of AVF in this study. The pressure was set at 50–70 mmHg to represent the mean arterial pressure in humans. This method had been used in several studies to investigate the effect of blood pressure on HUVECs and aortic endothelial cells, which increases angiogenesis and fibrosis (Tokunaga and Watanabe 1987; Tokunaga et al. 1989; Kato et al. 1994). Consistent with the previous studies, the results of this study confirmed that barometric pressure activates transitional signaling in endothelial cells and may be an approach to assess the blood pressure effect in cell experiments.

Compared with AVF induced via surgical anastomosis, aortocaval AVF created by needle puncture has no suture-related injury and forms overtly stenotic lesions less frequently due to the relatively large diameter of IVC. Several previous studies have reported an inflammation response and adverse remodeling of aortocaval AVF (Kuwahara et al. 2017; Matsubara et al. 2021; Sadaghianloo et al. 2017; Kudze et al. 2020). Therefore, regarding the effects of mechanical disturbance on AVF remodeling, aortocaval AVF may be an appropriate research model. Nevertheless, considering the potential differences in pathological changes between the aortocaval puncture and surgical anastomosis procedures, the findings of the study should be confirmed in other mouse AVF models or human AVF.

Pyrvinium pamoate is an approved anthelmintic drug with an inhibitory effect on β-catenin signaling, rather than a specific inhibitor of β-catenin activity. Nevertheless, it has been used to target β-catenin signaling in various oncology studies (Li et al. 2021; Polosukhina et al. 2017). In the present study, although the serum levels of pyrvinium pamoate were undetermined in the animal experiment, inhibition of β-catenin signaling was confirmed by the increased expression of axin2 in AVF. In the experiment testing the effect of pyrvinium on AVF thickening, a sham operated group treated with DMSO was lacking. As a result, it was not possible to assess the effect of DMSO on AVF thickening in this study. However, the experimental design used here is reasonable for investigating the effect of pyrvinium pamoate on AVF thickening.

## Conclusions

In conclusion, this study showed that mechanical disturbance in AVF activates β-catenin signaling in endothelial cells and induces the transition to myofibroblasts to cause the formation of AVF lesions. Although we showed that inhibition of β-catenin signaling attenuated AVF lesion formation, β-catenin signaling is not the only mechanism contributing to AVF lesions. An integrative strategy targeting more than one signaling pathway may be required to prevent AVF lesions.

## Supplementary Information

**Additional file 1.** Inhibition of β-catenin signaling attenuates arteriovenous fistula thickening in mice by suppressing myofibroblasts - Supplemental Information.

## Data Availability

The datasets analysed during the current study are available from the corresponding author on reasonable request.

## Abbreviations

AVF: Arteriovenous fistula
αSMA: Smooth muscle alpha-actin
CKD: Chronic kidney disease
IVC: Inferior vena cava
DMSO: Dimethyl sulfoxide
IHC: Immunohistochemistry
IF: Immunofluorescence
ITGB6: Integrin subunit β6
HUVEC: Human umbilical vein endothelial cell
GAPDH: Glyceraldehyde 3-phosphate dehydrogenase
