# Supplementary material for: Inhibition of β-catenin signaling attenuates arteriovenous fistula thickening in mice by suppressing myofibroblasts
Source: Mol Med. 2022 Jan 21;28:7. doi: 10.1186/s10020-022-00436-1 (PMC8783463; doi:10.1186/s10020-022-00436-1)
Supplement: Supplementary file 1 — Additional file 1. Inhibition of β-catenin signaling attenuates arteriovenous fistula thickening in mice by suppressing myofibroblasts - Supplemental Information. [file 10020_2022_436_MOESM1_ESM.docx]

**Inhibition of β-catenin signaling attenuates arteriovenous fistula thickening in mice by suppressing myofibroblasts – Supplemental Information**

**
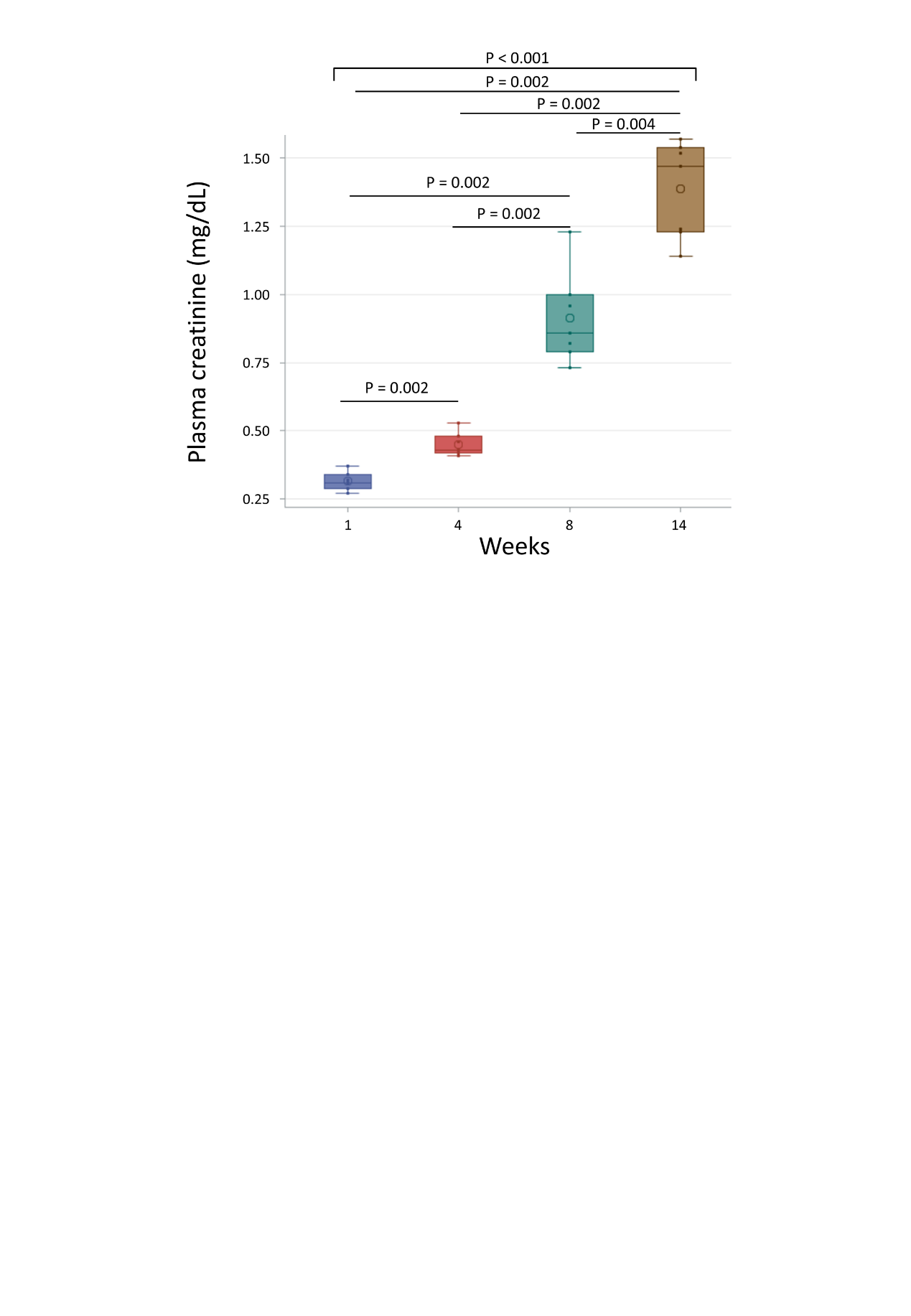
**

**Supplemental Figure 1. Adenine-induced renal impairment.** Plasma creatinine levels of mice receiving a 0.2% adenine diet at weeks 1, 4, 8, and 14. Data are presented as median, 1^st^ and 3^rd^ quartiles, maximal and minimal values in the box plots (n = 6). P-values were determined by the Wilcoxon signed rank-test with Bonferroni correction.

**
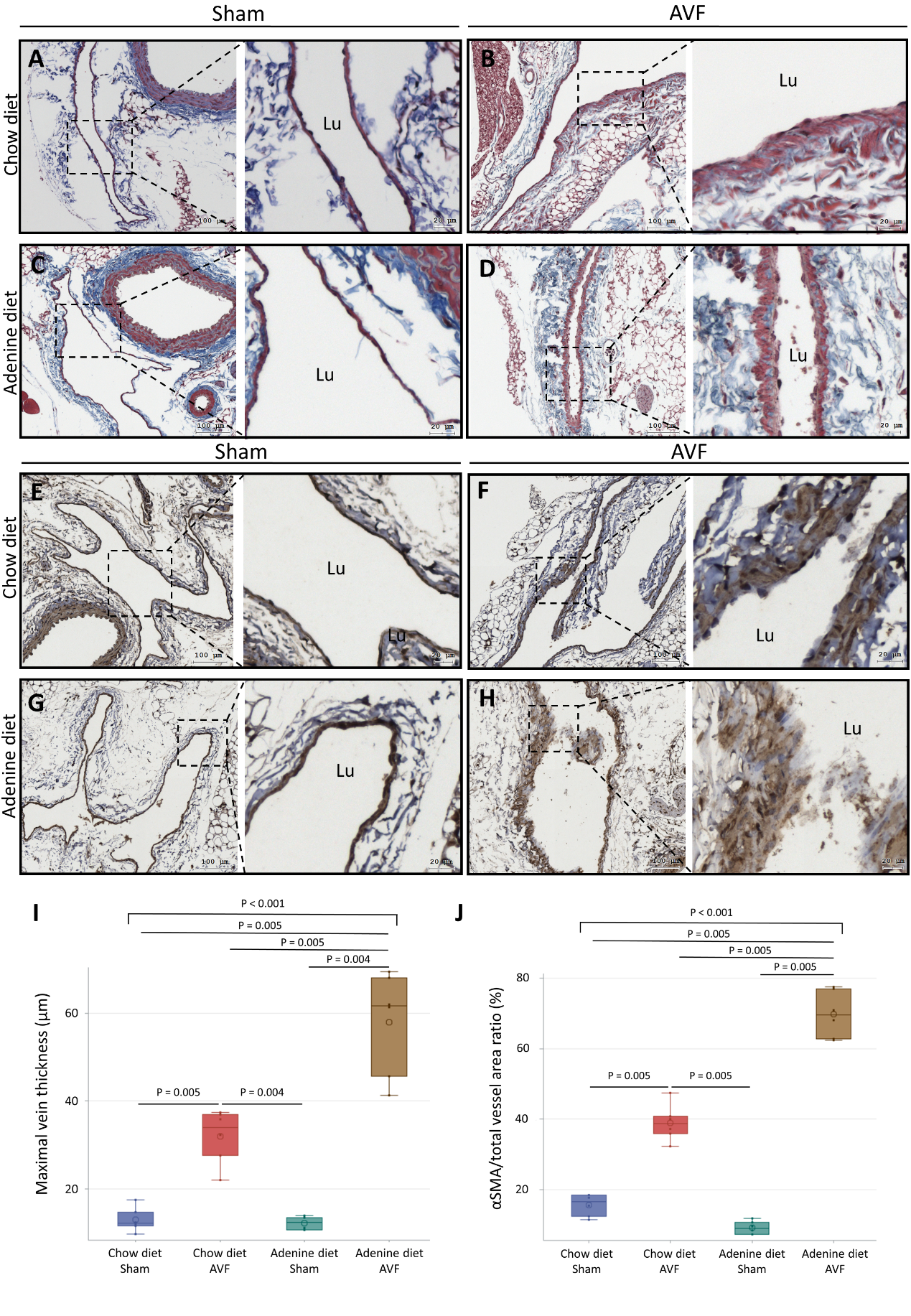
**

**Supplemental Figure 2. Effect of CKD on mouse AVF lesions.** The operation was performed 8 weeks after the chow diet or adenine diet was started. All data were obtained 6 weeks after the operation. **(A)–(D)** Masson’s trichrome stained sections. (**E)–(H)** The αSMA expression shown by IHC. (**I)** Quantification of vein thickness measured in Masson’s trichrome stained sections. **(J)** Quantification of αSMA expression measured in IHC sections. Data are presented as median, 1^st^ and 3^rd^ quartiles, maximal and minimal values in the box plots (n = 6 per group). P-values were determined by the Wilcoxon signed-rank test with Bonferroni correction. Insignificant P-values are not shown.

CKD, chronic kidney disease; AVF, arteriovenous fistula; IVC, inferior vena cava; IHC, immunohistochemistry; αSMA, smooth muscle alpha-actin; Lu, lumen.


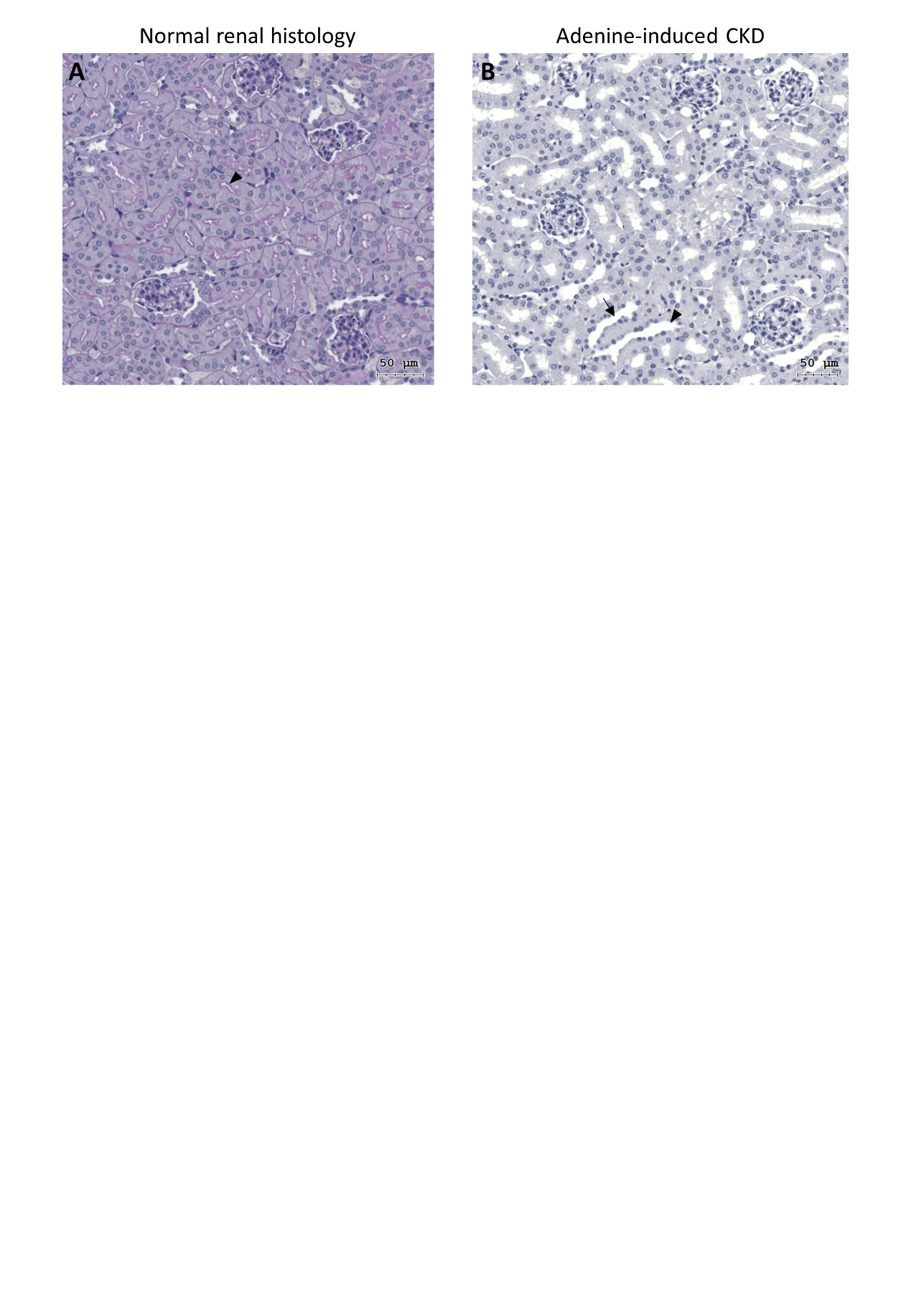


**Supplemental Figure 3. Adenine-induced CKD in mice.** **(A)** Normal renal histology from mice on a chow diet (PAS; arrowhead, intact brush border). **(B)** Renal histology from mice on a 0.2% adenine diet for 14 weeks (PAS stain; arrowhead, dilated renal tubule; arrow, thinning of renal tubular cells).

CKD, chronic kidney disease; PAS, Periodic Acid-Schiff stain.


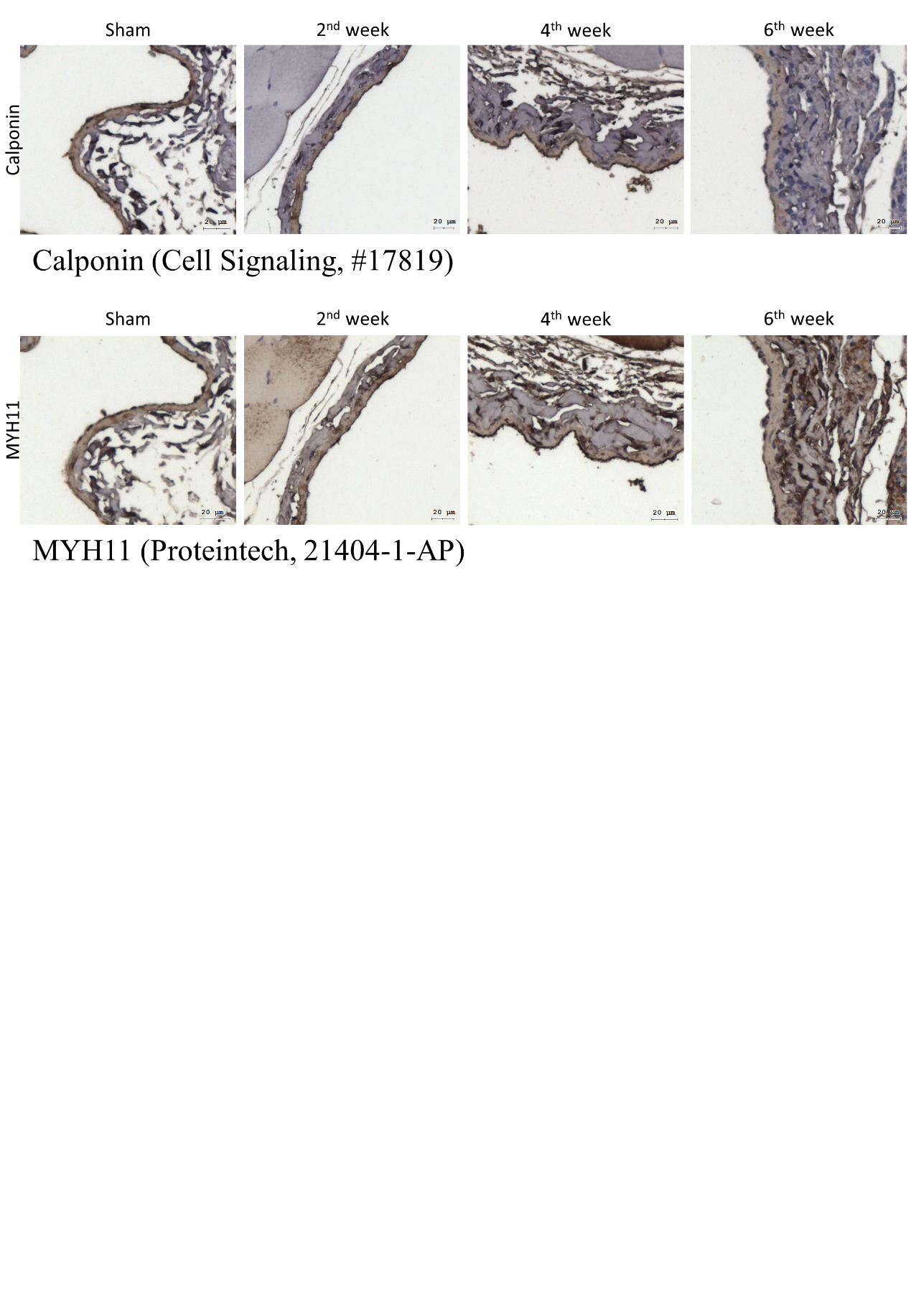


**Supplemental Figure 4. The expression of vascular smooth muscle cell marker calponin in mouse AVF.** Sham operated IVC specimens were obtained 6 weeks post-operation. AVF specimens were obtained at 2, 4, and 6 weeks post-operation. The specimens were stained for calponin by IHC.

AVF, arteriovenous fistula; IVC, inferior vena cava; IHC, immunohistochemistry.


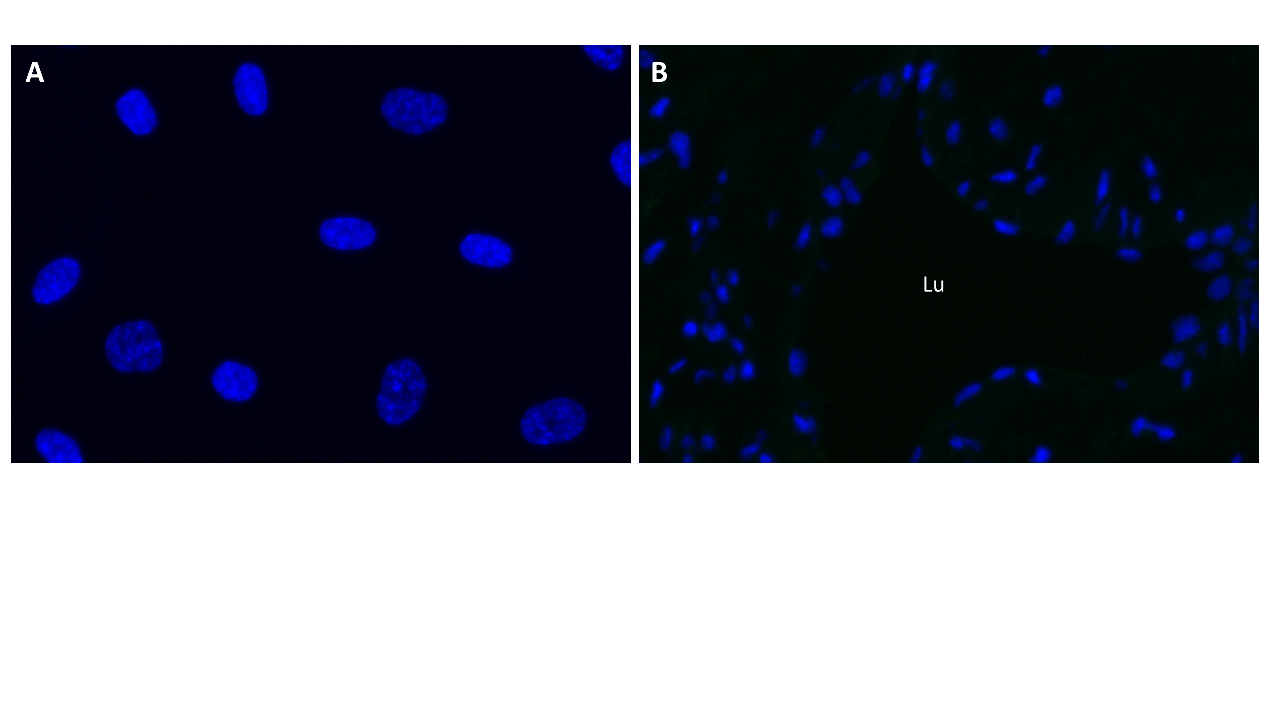


**Supplemental Figure 5. Image of isotype control primary antibody.** **(A)** HUVEC was exposed to increased barometric pressure and treated with the control solvent DMSO. (B) Tissue section of mouse AVF (Lu, lumen). Anti-CD20 (Abcam, ab64088) was used as the isotype control primary antibody. (secondary antibody: anti-rabbit antibody conjugated with Cy5, Abcam, ab97077) Nucleus was stained for dapi.

HUVEC, human umbilical veinous endothelial cell.


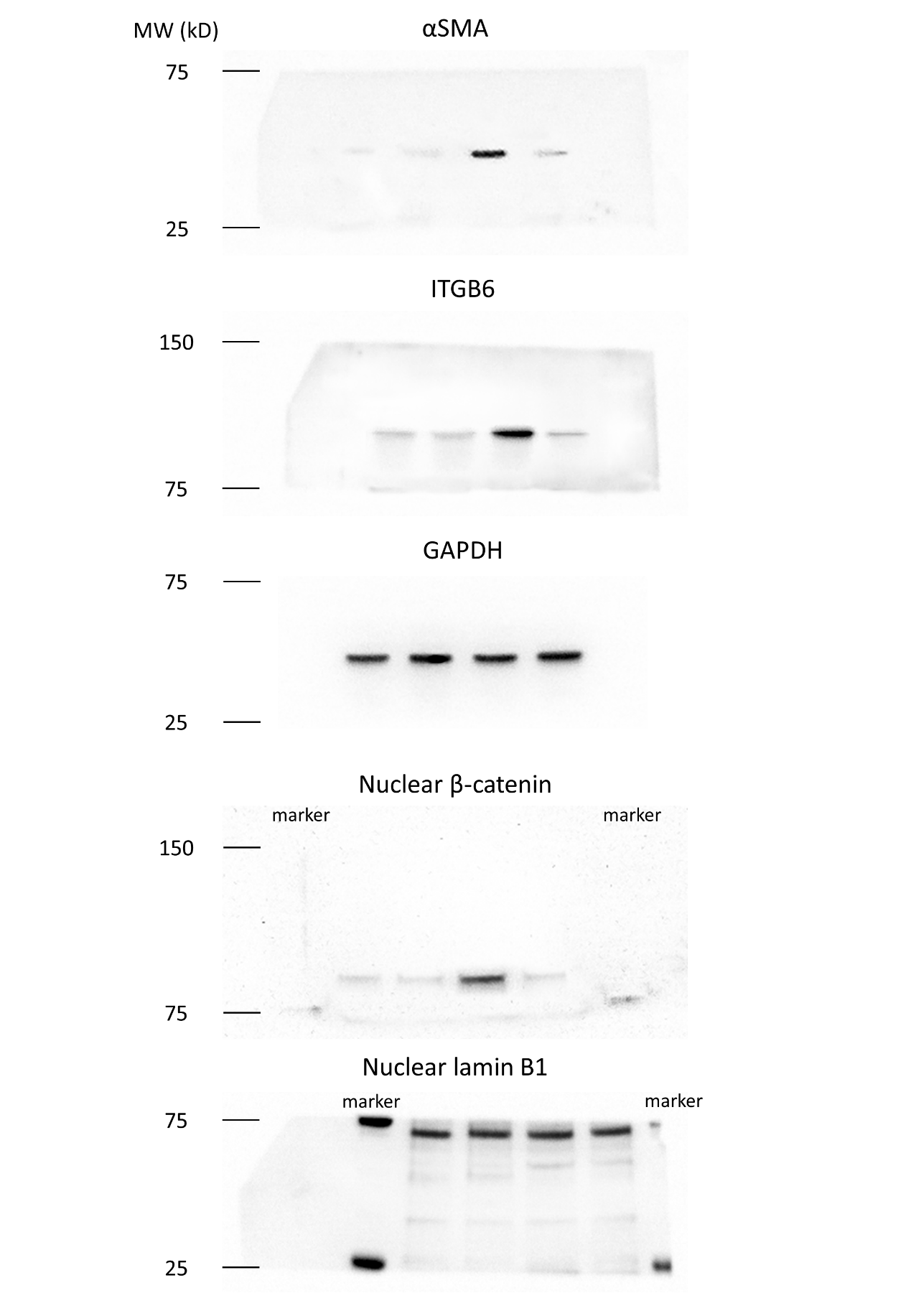


**Supplemental Figure 6. Uncut Western blots.**
